# The association of epicardial adipose tissue thickness and human neutrophil lipocalin with mitral annular calcification^☆^

**DOI:** 10.1016/j.ahjo.2025.100672

**Published:** 2025-11-12

**Authors:** Yeheng Xue, Xinyi Wang, Xiaohong Liu, Qingxue Zhang, Zhijian Liu, Bin Leng, Xiuchang Li

**Affiliations:** a Department of Echocardiography, The Second Affiliated Hospital of Shandong First Medical University, Taian, 271000, China; b Department of Cardiology, The Second Affiliated Hospital of Shandong First Medical University, Taian, 271000, China

**Keywords:** Mitral annular calcification, Epicardial adipose tissue, Human neutrophil lipocalin, Inflammatory response

## Abstract

**Study objective:**

Mitral annular calcification (MAC) is significantly associated with coronary artery stenosis and valvular dysfunction. And epicardial adipose tissue (EAT) can secrete inflammatory factors and human neutrophil lipocalin (HNL) are widely used as indicators of inflammation. There is now increasing evidence of a strong link between MAC and the inflammatory response. This study aims to investigate the effects of EAT thickness and HNL in MAC.

**Design:**

A cross-sectional approach was used in this study.

**Setting:**

The Second Affiliated Hospital of Shandong First Medical University.

**Participants:**

The included patients was classified into a calcification group (MAC group) and a control group based on the presence or absence of MAC on echocardiography.

**Interventions:**

None.

**Main outcome measures:**

The clinical data, HNL, EAT thickness of the two groups were measured, collected and analyzed. Logistic regression analysis was used to assess the independent risk factors for MAC and the receiver operating characteristic (ROC) curve was plotted to evaluate the effectiveness of EAT thickness and HNL in diagnosing MAC.

**Results:**

Patients in the MAC group had significantly higher basal and apical EAT thickness and HNL level than those in the control group. Basal EAT thickness was independently associated with MAC (OR = 2.003, 95 % CI = 1.474–2.721, *P* < 0.001). The AUC for basal EAT thickness to predict MAC was 0.880.

**Conclusion:**

Our data suggest EAT thickness and HNL were significantly associated with MAC, and basal EAT thickness near the right coronary artery was independently associated with MAC and had a high predictive value for MAC.

## Highlights

• A strong link between MAC and the inflammatory response
• Correlation between MAC and EAT thickness was not consistent at different sites
• There are few published reports regarding the association between HNL and EAT thickness or valve calcification

## Graphical abstract

**Figure:**
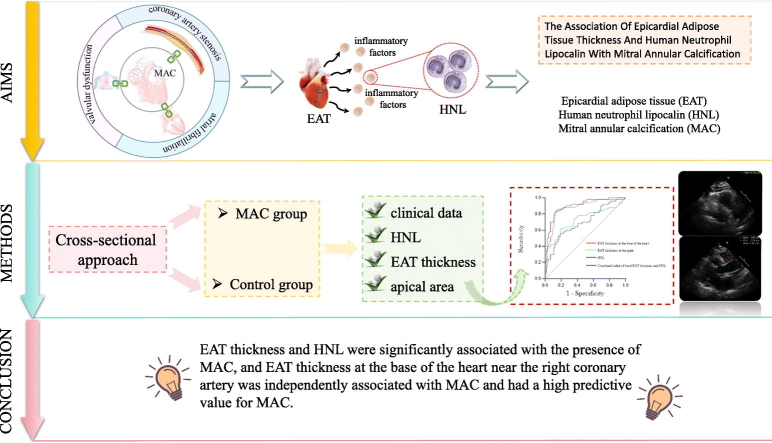

## 1 Introduction

Mitral annular calcification (MAC) is a common finding on cardiovascular imaging and is associated with cardiovascular morbidity and mortality and adverse outcomes of cardiovascular procedures and surgery [1,2]. Risk factors for MAC include age, smoking, hypertension, diabetes mellitus and dyslipidaemia; increased mitral valve pressure due to aortic stenosis, abnormal calcium and phosphorus metabolism, and inflammation may also lead to valve calcification [3]. In addition to C-reactive protein (CRP), a significant association between the inflammatory marker interleukin-6 (IL-6) and MAC has been demonstrated [3,4]. However, the mechanism of MAC remains unclear, which needs further investigation.

Epicardial adipose tissue (EAT) is located between the myocardium and the visceral pericardium [5].Under pathological conditions, EAT can lead to the development and progression of cardiovascular disease through vascular secretion or paracrine pathways [6,7]. EAT is not uniformly distributed in the heart, and the inflammatory activity of EAT varies at different sites, so that an imbalance between the secretion of anti-inflammatory and pro-inflammatory EAT adipose factors has a significant impact on the progression of cardiovascular disease, including valvular calcification. Human neutrophil lipocalin (HNL) can be stored as a pre-formed protein in secondary granules of neutrophils and released by neutrophils upon stimulation [8,9], and HNL is currently being used as a potential clinical biomarker for inflammatory diseases. However, there were fewer studies on EAT thickness and HNL in annular calcification and the aim of our study was to investigate the relationship between EAT thickness and HNL and MAC.

## 2 Materials and methods

Patients admitted to the Second Affiliated Hospital of Shandong First Medical University between 2022 and 2024 were selected for the study. All included patients had echocardiographic images of a high quality, and the clinical data were complete and the patients were divided into a calcification group and a control group based on the presence or absence of MAC on echocardiography. The study was approved by the hospital ethics committee (ethics approval number: 2022-89) and informed consent was obtained from the patients. Exclusion criteria included patients with severe underlying diseases associated with acute and chronic systemic inflammatory reactions, hypercalcaemia and hyperphosphatemia. Patients with congenital mitral valve disease or prosthetic valves, rheumatic heart disease or cardiomyopathy, poor echocardiographic window and incomplete clinical data were also excluded from the study. Diabetes mellitus was defined as the guidelines of the American Diabetes Association [10]. Hypertension was defined as BP ≥ 130/80 mmHg or receiving antihypertensive therapy [11].

### 2.1 Blood sample analysis

Fasting blood samples were collected on the day of admission or the next day in both groups and sent for examination to obtain hematological indicators. Biochemical indicators including blood cell count, blood lipids, liver and kidney functions were determined by professional staff of the Department of Laboratory Medicine using relevant professional instruments. HNL was detected by enzyme-linked immunosorbent assay with a reference interval of 0–115.55 μg/L using a kit supplied by Changchun Bode Bio-Technology Co.

### 2.2 Echocardiography examination

**Fig. 1:**
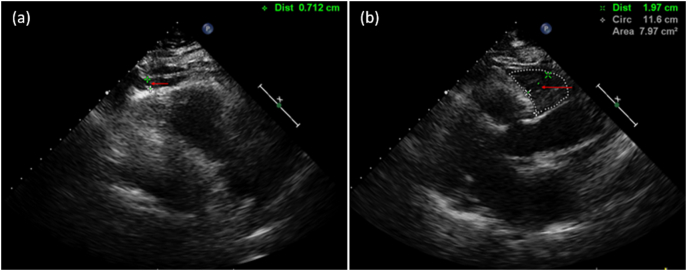
Parasternal long-axis section of the left ventricle showing epicardial adipose tissue in the apex and bottom of the heart (arrow points). (a). EAT thickness near the apex. (b) EAT thickness near the heart base. EAT: epicardial adipose tissue.

Transthoracic echocardiography was performed on each subject using a PHILIPS Epiq 7c color Doppler echocardiograph (equipped with an S5-1 probe at 1–5 MHz, Philips, Netherlands), and the examiner was blinded to the clinical data. Data including left atrial (LA) diameter, left ventricular end diastolic diameter (LVEDD), right ventricular(RV) diameter and left ventricular ejection fraction (LVEF) were measured by two-dimensional echocardiography. The presence and degree of regurgitation in each valve was assessed by Doppler color flow mapping. The presence or absence of MAC was also evaluated. MAC is defined as an echocardiographically visible structure that appears irregular and rough, exhibiting strong echogenicity. It is located between the left atrium and the left ventricle, identifiable in the parasternal long-axis or short-axis views at the level of the mitral annulus, as well as in apical four-chamber, three-chamber, or two-chamber views [12]. EAT thickness was assessed in a parasternal view, perpendicular to the right ventricular free wall. This involved measuring the thickness of adipose tissue located between the right ventricular free wall and the adjacent pericardium at both apical and basal regions of the heart (the thickest portion of the right ventricular free wall anterior to the atrioventricular/coronary sulcus) during end-systole. The measurements were averaged over three consecutive end-systolic heart cycles (Fig. 1) [13].

### 2.3 Statistical analysis

Measurements that followed a normal distribution were presented as mean ± standard deviation, and the independent samples *t*-test was employed. Measurements that did not conform to a normal distribution were reported as median (interquartile range) [M (P25, P75)], utilizing non-parametric tests such as the Wilcoxon rank-sum test. Categorical data were expressed as percentages, with the χ^2^ test or Fisher's exact test applied accordingly.

Univariate and multivariate logistic regression analyses were conducted to identify risk factors for MAC. The variables were selected for multivariable analysis based on univariable *p* < 0.05. Receiver Operating Characteristic (ROC) curves were generated for EAT thickness and HNL in diagnosing MAC, from which cut-off values, area under the curve (AUC), sensitivity, and specificity for each index were calculated. A two-sided α = 0.05 was established as the significance level for all statistical analyses. All aforementioned analyses were performed using SPSS version 26.0 for data analysis purposes.

## 3 Results

### 3.1 Baseline and blood variables of the study and control groups

A total of 124 patients were included in the study, including 50 patients (40.3 %) in the MAC group and 74 patients (59.7 %) in the control group. There were no statistically significant differences in age, sex, BMI, proportion of patients with hypertension and diabetes mellitus between the two groups. The HNL levels in the MAC group were significantly elevated compared to those in the control group, measuring 107.12 (49.17–145.2) ng/mL versus 61.58 (35.82–99.04) ng/mL, respectively. Concurrently, the leukocyte count, neutrophil count and red cell distribution width (RDW) were significantly elevated in the MAC group compared to the control group (*P* < 0.05). In contrast, the remaining hematological indices did not show statistically significant differences between the two groups (*P* > 0.05), as illustrated in Table 1.

**Table 1:** Baseline variables of patients with and without MAC.

Variables	Controls (*n* = 74)	MAC (*n* = 50)	t/Z/x^2^	*P* value
Age (years)	69.47 ± 8.39	70.74 ± 8.50	−0.820	0.414
Gender				
Male, n (%)	29 (39.2)	16 (32.0)	0.667	0.414
Female, n (%)	45 (60.8)	34 (68.0)		
Hypertension, n (%)	54 (73.0)	41 (82.0)	1.357	0.244
Diabetes mellitus, n (%)	22 (29.7)	23 (46.0)	3.42	0.065
BMI (kg/m^2^)	25.03 ± 3.41	26.11 ± 3.75	−1.677	0.096
Medicine use				
ACEI/ARB, n (%)	50 (67.6)	38 (76.0)	1.030	0.310
CCB, n (%)	45 (60.8)	32 (64.0)	0.129	0.720
Statin, n (%)	47 (63.5)	39 (78.0)	2.946	0.086
SGLT2, n (%)	9 (12.2)	9 (18.0)	0.820	0.365
GLP1, n (%)	4 (5.4)	5 (10.0)	0.378	0.539
Biochemical parameters				
Hemoglobin (g/dL)	135.00 (126.00–144.75)	133.23 (119.00–146.00)	−0.805	0.421
WBC (×10^9^/L)	5.63 (4.64–6.49)	6.52 (5.04–7.97)	−2.858	0.004
Platelet count (×10^9^)	210.27 ± 47.72	217.08 ± 52.91	−0.746	0.457
RDW (%)	12.50 (12.10–13.20)	12.90 (12.40–13.63)	−2.236	0.025
Neutrophil count (×10^9^/L)	3.41 (2.76–4.02)	4.25 (3.20–5.33)	−3.145	0.002
ALT (U/L)	17.00 (13.00–22.00)	17.89 (13.75–23.00)	−0.339	0.734
AST (U/L)	20.00 (16.00–22.25)	17.50 (15.00–23.25)	−0.896	0.370
eGFR (mL/min)	91.96 (86.79–100.95)	90.89 (84.54–99.09)	−1.003	0.316
Creatinine (μmol/L)	58.30 (49.23–68.00)	57.75 (47.93–70.05)	−0.046	0.963
Triglyceride (mmol/L)	1.29 (0.98–1.64)	1.47 (0.98–2.23)	−1.480	0.139
HDL-cholesterol (mmol/L)	1.23 (1.01–1.49)	1.14 (0.99–1.39)	−1.375	0.169
LDL-cholesterol (mmol/L)	2.32 (1.86–3.02)	2.41 (1.82–3.37)	−0.413	0.680
HNL (ng/mL)	61.32 (34.32–96.08)	110.57 (52.20–155.52)	−3.744	<0.001

MAC: mitral annular calcification, BMI: body mass index, ACEI: angiotensin converting enzyme inhibitor, ARB: angiotensin receptor blocker, CCB: calcium channel blocker, SGLT2: sodium glucose cotransporter 2, GLP1: glucagon-like peptide-1, WBC: white blood cell, RDW: red cell distribution width, AST: aspartate aminotransferase, ALT: alanine aminotransferase, eGFR: estimated glomerular filtration rate, LDL-cholesterol: low density lipoprotein cholesterol, HDL-cholesterol: high density lipoprotein cholesterol, HNL: human neutrophil lipocalin.

### 3.2 Echocardiographic variables of the study and control groups

When compared to controls, individuals in the MAC group exhibited larger LA diameter, LVEDD, LVPW thickness, E/e', and a lower LVEF. Furthermore, the thickness of EAT at both the base and apical regions of the heart was markedly greater in patients within the MAC group than those in the control group. Additionally, diastolic dysfunction was observed in 98.0 % of patients within the MAC group—significantly higher than that seen in the control population at 85.1 %, as presented in Table 2.

**Table 2:** Echocardiographic variables of patients with and without MAC.

Variables	Controls (*n* = 74)	MAC (*n* = 50)	t/Z/x^2^	P value
EAT_Basal_ (mm)	10.62 ± 1.95	13.85 ± 2.02	−8.936	<0.001
EAT_Apex_ (mm)	5.93 ± 1.39	7.58 ± 2.32	−4.503	<0.001
Mean EAT_Basal_ and EAT_Apex_ (mm)	8.27 ± 1.40	10.72 ± 1.68	−8.783	<0.001
LA (mm)	39.83 ± 3.73	43.93 ± 4.28	−5.660	<0.001
IVS (mm)	10.13 ± 0.72	10.67 ± 1.17	−2.921	0.005
LVEDD (mm)	46.05 ± 3.72	49.56 ± 4.77	−4.597	<0.001
LVPW (mm)	10.00 (9.50–10.40)	10.30 (9.85–10.80)	−2.553	0.011
LVEF (%)	64.65 (61.90–68.03)	61.15 (58.73–64.95)	−3.398	<0.001
LVFS (%)	35.50 (33.38–37.93)	33.35 (31.48–35.98)	−3.016	0.003
E wave	70.80 (57.85–88.13)	77.50 (63.33–97.55)	−1.658	0.097
A wave	97.18 ± 17.36	101.23 ± 22.11	−1.140	0.256
E/A	0.70 (0.60–0.83)	0.76 (0.61–1.00)	−0.983	0.326
e'	4.65 (3.93–5.32)	4.40 (3.78–5.20)	−0.818	0.413
E/e’	16.05 (12.13–18.09)	16.64 (14.00–23.89)	−2.139	0.032
LVDD, n (%)	63 (85.1 %)	49 (98.0 %)	4.274	0.039
MR (more than mild), n (%)	20 (26.0 %)	27 (51.9 %)	9.024	0.003
TR (more than mild), n (%)	12 (15.6 %)	18 (34.6 %)	6.299	0.012
AR (more than mild), n (%)	31 (40.3 %)	18 (34.6 %)	0.420	0.517

MAC: mitral annular calcification, EAT: epicardial adipose tissue, LA: left atrium, IVS: interventricular septum, LVEDD: left ventricular end diastolic diameter, LVPW: left ventricular posterior wall, LVEF: left ventricular ejection fraction, LVFS: left ventricular fractional shortening, E wave: pulse Doppler transmitral early velocity; A wave: pulse Doppler late transmitral peak flow velocity; e′: early diastolic mitral annular velocity; LVDD: left ventricular diastolic dysfunction; MR: mitral regurgitation; TR: tricuspid regurgitation, AR: aortic regurgitation.

### 3.3 Regression analysis and ROC curve for predicting MAC

**Fig. 2:**
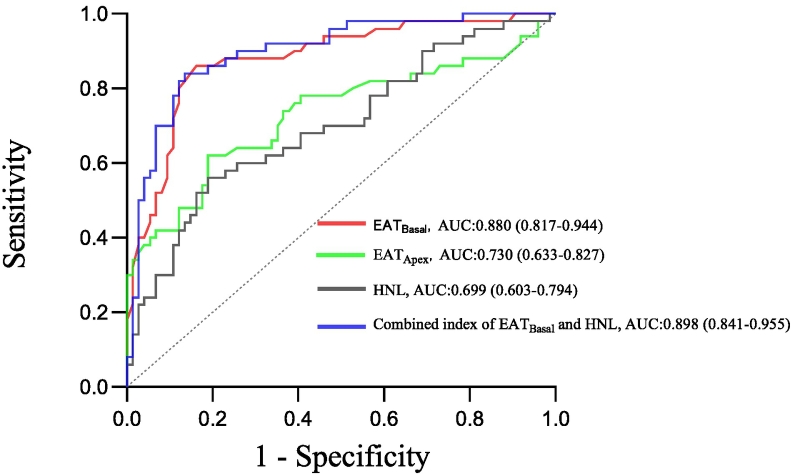
Receiver operating characteristic (ROC) curves to predict MAC. MAC: mitral annular calcification, EAT: epicardial adipose tissue, HNL: human neutrophil lipocalin.

A logistic regression analysis was performed to ascertain independent associations among various variables with respect to MAC development. Collinearity analysis results indicated that the Variance Inflation Factor (VIF) of all the independent variables was <5, suggesting no significant collinearity among them, as shown in Table 3. In multifactorial regression, after adjusting for the effects of leukocytes, RDW, triglycerides, and EAT thickness in the basal and apical parts of the heart, HNL was not found to be an independent risk factor for MAC. However, among these factors, EAT thickness at the base of the heart emerged as an independent risk factor for MAC progression (OR = 2.003; 95 % CI = 1.474–2.721; *P* < 0.001), as detailed in Table 4.The results of the ROC curve showed that the cut-off value of EAT thickness at the base of the heart for predicting MAC was 12.35 mm, with an AUC of 0.880, and the sensitivity and specificity were 86.0 % and 83.8 %, respectively; whereas the cut-off value of HNL for predicting MAC was 103.38 ng/mL, with an AUC of 0.699, and the sensitivity and specificity were 56.0 % and 81.1 %, respectively, and the combination of the two factors increased the predictive value, with an AUC of 0.898, and the sensitivity and specificity were 84.0 % and 86.5 %, respectively, as shown in Fig. 2.

**Table 3:** Collinear statistics of the included binary Logistic regression variables.

Variables	Collinearity statistics	
	Tolerance	VIF
WBC	0.827	1.210
RDW	0.933	1.072
Triglyceride	0.961	1.041
HNL	0.827	1.209
EAT basal	0.726	1.378
EAT apex	0.761	1.315

MAC: mitral annular calcification, WBC: white blood cell, RDW: red cell distribution width, EAT: epicardial adipose tissue, HNL: human neutrophil lipocalin.

**Table 4:** Univariate regression and multivariate regression analysis of patients with and without MAC.

Variables	Univariate regression coefficient			Multivariate regression coefficient		
	OR (95 % CI)	β	P value	OR (95 % CI)	β	P value
Age	1.018 (0.975–1.064)	0.018	0.411			
Gender	0.730 (0.343–1.555)	−0.314	0.415			
BMI	1.091 (0.984–1.211)	0.087	0.099			
Hypertension	1.687 (0.696–4.089)	0.523	0.247			
Diabetes mellitus	2.013 (0.954–4.249)	0.700	0.066			
WBC	1.381 (1.108–1.721)	0.323	0.004	1.156 (0.859–1.555)	0.070	0.338
RDW	1.573 (1.015–2.436)	0.453	0.042	1.089 (0.552–2.147)	0.085	0.807
eGFR	0.982 (0.957–1.006)	−0.019	0.146			
Triglyceride	1.531 (1.009–2.322)	0.426	0.045	1.531 (0.897–2.612)	0.426	0.118
HDL-cholesterol	0.332 (0.091–1.217)	−1.101	0.096			
LDL-cholesterol	1.189 (0.762–1.855)	0.173	0.445			
HNL	1.010 (1.003–1.016)	0.010	0.002	1.007 (0.999–1.015)	0.007	0.081
EAT_Basal_	2.326 (1.727–3.134)	0.844	<0.001	2.003 (1.474–2.721)	0.694	<0.001
EAT_Apex_	1.646 (1.299–2.086)	0.498	<0.001	1.329 (0.979–1.806)	0.285	0.069

MAC: mitral annular calcification, BMI: body mass index, WBC: white blood cell, RDW: red cell distribution width, EAT: epicardial adipose tissue, eGFR: estimated glomerular filtration rate, LDL-cholesterol: low density lipoprotein cholesterol, HDL-cholesterol: high density lipoprotein cholesterol, HNL: human neutrophil lipocalin.

## 4 Discussion

In the present study, we found that HNL and EAT thickness in the basal and apical regions of the heart were significantly higher in the MAC group compared to the control group. Furthermore, EAT thickness in the basal region was independently associated with the development of MAC. As a marker with substantial clinical and prognostic value, several studies have shown that MAC is associated with the incidence of cardiovascular diseases such as myocardial infarction, stroke and atrial fibrillation, as well as mortality [[12], [13], [14]]. The pathophysiological mechanism of MAC remains inadequately understood; however, one critical process involved in annular calcification is the transformation of valvular myofibroblasts into osteoblasts. Additionally, factors associated with atherosclerosis—such as valvular endothelial rupture and inflammatory infiltration—play a pivotal role in facilitating this cellular conversion [3,15]. In this study, we identified a significant association between MAC and LVDD. This association which may be attributed to elevated ventricular filling pressures, asynchronous contraction of the left ventricular wall, and abnormal atrial systolic function caused by rapid heart rate [16]. The robust association between MAC and LVDD could also stem from common risk factors such as age, hypertension, diabetes and other chronic diseases, as well as mitral valve dyskinesia due to calcification of the mitral annulus extending into the orifice region or leaflets, leading to increased ventricular loading and consequent diastolic dysfunction.

EAT is mainly located in the atrioventricular and interventricular grooves and distributed along the coronary arteries, and can be divided into pericoronary EAT and perimyocardial EAT [17]. Lipocalin secreted by epicardial adipocytes improves endothelial function and mitigates oxidative stress by stimulating nitric oxide synthase and indirectly reduces IL-6 and CRP levels by decreasing tumour necrosis factor-α production [18]. However, an increase in the thickness or volume of EAT may result in macrophage and T lymphocyte infiltration, leading to excessive secretion of inflammatory mediators such as IL-1 and IL-6. This process can subsequently diminish lipocalin secretion. A study by Mazurek T et al [19] confirmed the presence of an extensive inflammatory response in the EAT of CAD patients, with significantly increased levels of inflammatory and chemokine expression in the EAT compared to subcutaneous adipose tissue.

The association between MAC and EAT thickness has been discovered in current research. Nabati M et al. [20] classified patients into four classes based on the extent of calcification involving the mitral annulus and found that the likelihood and severity of MAC was higher in the thicker EAT group, however, no differences were observed in the prevalence of moderate versus severe MAC between groups classified by EAT thickness. Similarly, Guler S et al [21] demonstrated that EAT thickness was significantly higher in patients in the MAC group than in the control group and that EAT thickness was positively correlated with the presence of MAC. And Argan O et al [22] suggest that EAT is an independent predictor for the presence of severe MAC in patients aged ≥60 years. Nevertheless, the underlying mechanism linking EAT to MAC remains unclear. Firstly, as a component of visceral obesity, EAT is closely associated with cardiovascular risk factors such as hypertension, hyperlipidaemia, diabetes mellitus and metabolic syndrome, which may contribute to the development and progression of MAC. Secondly, unlike other visceral adipose tissues, EAT, due to its anatomical proximity to the heart and the lack of isolation of the fascia from the heart, may influence the coronary arteries and the cardiac valves through paracrine or vascular secretion mechanism involving pro-inflammatory adipokines.

Originally discovered and purified from human neutrophils, HNL can be pre-stored in neutrophil-specific granules from which it is readily released upon cell activation. The predominant form isolated from neutrophils is a homodimer with a molecular weight of 45 kDa; however, monomeric (24 kDa) and heterodimeric (>90 kDa) molecular forms were later found to exist [23]. Currently, there are no published reports regarding the association between HNL and EAT thickness or valve calcification. In previous studies, HNL has frequently been utilized as an indicator of infection, with comparatively fewer investigations focusing on its role in cardiovascular disease beyond diagnosing associated infectious conditions [24,25].

In our study, we found for the first time that HNL was associated with EAT thickness and MAC. In multifactorial regression, HNL was not found to be independently associated with the presence of MAC. Given the relatively small sample size in this study, we cannot dismiss the possibility that HNL was independently associated with the presence of MAC while expanding the sample. The results from ROC curve analysis demonstrated that HNL had an optimal cut-off value of 103.38 ng/mL, with an area under the curve of 0.699, a sensitivity and specificity of 56.0 % and 81.1 % respectively, and a higher value for predicting MAC in conjunction with EAT thickness at the base of the heart. These findings contribute to existing research on HNL within cardiovascular disease contexts and suggest the potential relevance of HNL in the study of annular calcification.

## 5 Conclusion

Our findings found that EAT thickness and HNL were significantly associated with the presence of MAC, and EAT thickness at the base of the heart near the right coronary artery was independently associated with MAC and had a high predictive value for MAC.

### 5.1 Limitation

First, the present study is a cross-sectional study that could not directly confirm the causal relationship between EAT thickness and the occurrence of MAC, and future prospective studies are needed. Second, the relatively small sample of this study limited our ability to grade the degree of annular calcification.

## CRediT authorship contribution statement

**Yeheng Xue:** Writing – review & editing, Writing – original draft, Project administration, Methodology, Formal analysis, Conceptualization. **Xinyi Wang:** Writing – review & editing, Writing – original draft, Supervision, Investigation, Data curation, Conceptualization. **Xiaohong Liu:** Visualization, Investigation, Formal analysis. **Qingxue Zhang:** Validation, Resources, Data curation. **Zhijian Liu:** Investigation, Data curation, Conceptualization. **Bin Leng:** Resources, Investigation, Formal analysis. **Xiuchang Li:** Supervision, Project administration, Methodology, Conceptualization.

## Ethical statement

All procedures were performed in compliance with relevant laws and institutional guidelines, and the study was approved by the hospital ethics committee (ethics approval number: 2022–89, data: 2022-09-23) and informed consent was obtained for experimentation with human subjects.

## Declaration of competing interest

This research did not receive any specific grant from funding agencies in the public, commercial, or not-for-profit sectors and all authors declare that they have no conflict of interest.

## Data Availability

Our research data includes confidential information including patient information, and readers could contact the corresponding author if they require the full dataset.
